# The complete chloroplast genome sequence of the *populus* cultivar “*Populus × Beijingensis*”: a type of unique Chinese *populus*

**DOI:** 10.1080/23802359.2025.2559715

**Published:** 2025-09-16

**Authors:** Yong-Hao Nan, Yu-Rong Ren, Jian-Ming Shi, Kang-Zhong Wang, Jian-Rong Wei, Ji-Gang Li

**Affiliations:** College of Life Sciences, Hebei University, Baoding, China

**Keywords:** Chloroplast genome, populus, phylogeny analysis

## Abstract

This study examines the chloroplast genome of *Populus × beijingensis*, a deciduous tree species from the genus *Populus* in the *Salicaceae* family, which is highly valued for its ecological and economic significance. The sequencing result shows that the chloroplast genome of *P.×beijingensis* is approximately 157,070 base pairs (bp) in length, with the large single-copy (LSC) region being 85,192 bp and the small single-copy (SSC) region at 16,564 bp. It encodes a total of 128 genes, comprising 84 protein-coding genes, 36 transfer RNA (tRNA) genes, and 8 ribosomal RNA (rRNA) genes. This research establishes a foundation for future studies on the genetic diversity and ecological adaptation of *P.×beijingensis* and other *Populus* species.

## Introduction

*Populus*, commonly known as poplar, is widely distributed in temperate and subtropical regions of the Northern Hemisphere (Liao et al. 2023). These trees are recognized for their rapid growth, ease of cultivation, and significant ecological and economic contributions (Wan et al. 2023). Among them, *Populus × beijingensis* Linaeus 1956 (Wang and Dong 1982) was artificially hybridized by the Institute of Forestry Science, Chinese Academy of Forestry in 1956 and is extensively cultivated in China due to its adaptability to various climates and altitudes.

The chloroplast genome of a tall tree is a valuable genetic resource that plays a crucial role in photosynthesis and chloroplast function (Li et al. 2024; Mao et al. 2024). In plant systematics, the relatively conserved nature and appropriate evolutionary rate of chloroplast genomes make them ideal tools for constructing accurate phylogenetic trees and detecting targets of natural hybridization (Birky 1995). Recent studies have indicated that poplar chloroplast genomes are highly conserved, featuring a typical quadrilateral structure with few variable regions. This conservation positions them as powerful tools for examining phylogenetic relationships and genetic diversity within the genus (Ke et al. 2022; Gu et al. 2024). In this study, we analyzed the chloroplast genome of *P.×beijingensis* to provide insights into its genetic characteristics and evolutionary position. This work not only enhances our understanding of *P.×beijingensis* but also contributes to a broader comprehension of poplar species and their potential applications in forestry and ecological restoration.

## Materials and methods

**Figure 1.**
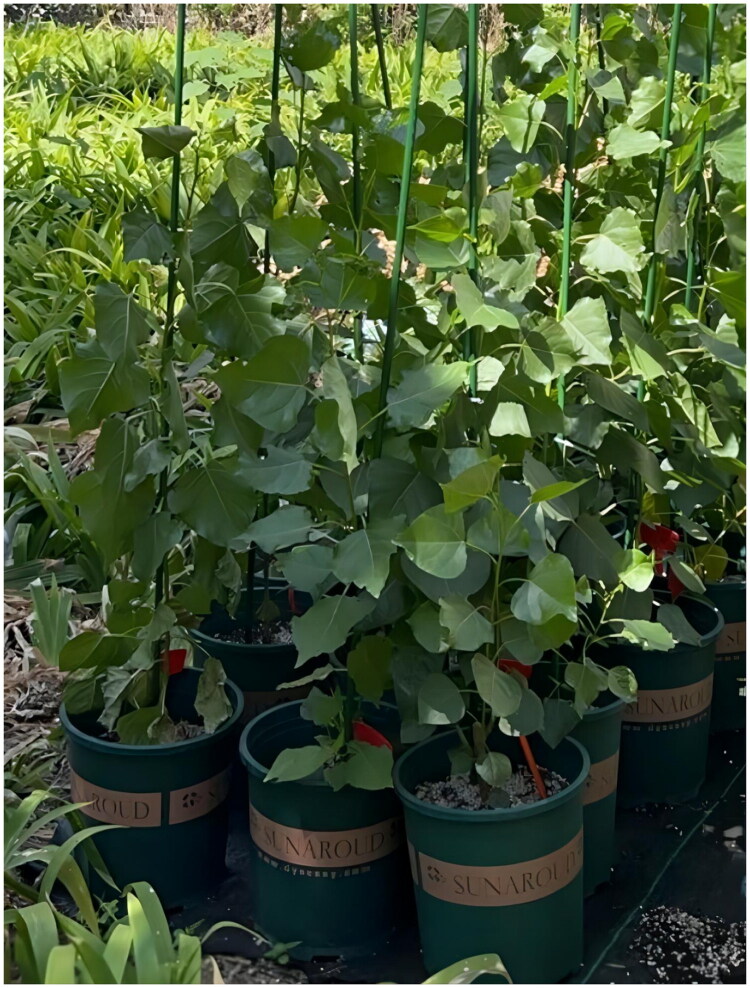
Pictures of *Populus × beijingensis*’ potted plants. The photo was taken by Yu-Rong Ren at the college of life sciences, Hebei university. Leaf blade margin undulating ruffled coarsely serrate, translucent margin, with sparse margin hairs, posterior smooth.

Shoots for cuttings of *P.×beijingensis* were collected from the Chinese Academy of Forestry (106°32′E, 40°01′N). After collection, the cuttings were transported to Hebei University, Baoding, Hebei Province. Under soil culture conditions in the laboratory, the cuttings successfully germinated and produced leaves (Figure 1). After collecting fresh leaves, the remaining specimens were stored in the Laboratory of Plant-Insect Interaction Ecology, Hebei University (contact person: Wei Jian-Rong, jrwei9@126.com), specimen number: HBSK402-BJY.

Fresh leaves were collected for DNA extraction using a spin column-based commercial kit (CW2298S, CWBIO, China) following the manufacturer’s instructions. The DNA concentration and integrity were assessed using a Nanodrop spectrophotometer (ThermoFisher Scientific Inc., Waltham, MA, USA) (Versmessen et al. 2024). Qualified DNA samples were then sent to Applied Protein Technology (Shanghai, China) for library construction and sequencing with an Illumina NovaSeq-6000 system (Illumina Inc., San Diego, CA). The sequencing mode is 150 bp PE. A total of 21.81 GB of raw data and 21.33 GB of filtered, clean data were generated (Cock et al. 2010). The chloroplast genome was assembled using SPAdes v3.10.1 (k 21,77,127) (Dmitry et al. 2020). The accuracy of the annotated genome was evaluated with the gbcheak subcommand of CPStools (Huang et al. 2024). The chloroplast genome sequence of *Populus trichocarpa* (GenBank: MW376841) (Wang et al. 2022) served as a reference for manually comparing and adjusting the genome of *P.×beijingensis* using Geneious (Wang et al. 2024) to ensure its accuracy and integrity.

MAFFT v7.526 (Katoh et al. 2019) and Gblocks (Leduc et al. 2018) were used to perform sequence alignment and trim the conserved regions. Using IQ-tree v2.4.0 (Nguyen et al. 2015) and FigTree v1.4.4 with *Betula utilis* as the outgroup, a maximum likelihood phylogenetic tree was constructed for *P.*×*beijingensis* and 15 other *Populus*. Using the SH-aLRT and UFBoot algorithms, each was repeated 1000 times. The nucleotide substitution model used for sequence alignment is the Bayesian Information Criterion (BIC) model K3Pu + F+R4. Using CPGview online website (http://www.1kmpg.cn/cpgview) mapped the *P.*×*beijingensis* chloroplast genome. SAMtools (Li et al. 2009) and Excel tables were used to map the chloroplast genome coverage depth of *P.×beijingensis.* The junction site information of the chloroplast genome tetrad structure was generated by the Genepioneer’s bioinformatics platform (Nanjing, China). Sequence alignment was performed with VISTAtools (Zhang et al. 2024) for the complete chloroplast genomes of *P.×beijingensis*, *Populus trichocarpa* (GenBank: MW376841), *Populus tomentosa* (GenBank: NC_040866), and *Populus alba* var*. Pyramidalis* (GenBank: NC_040929) (Zong et al. 2019).

## Results

**Figure 2.**
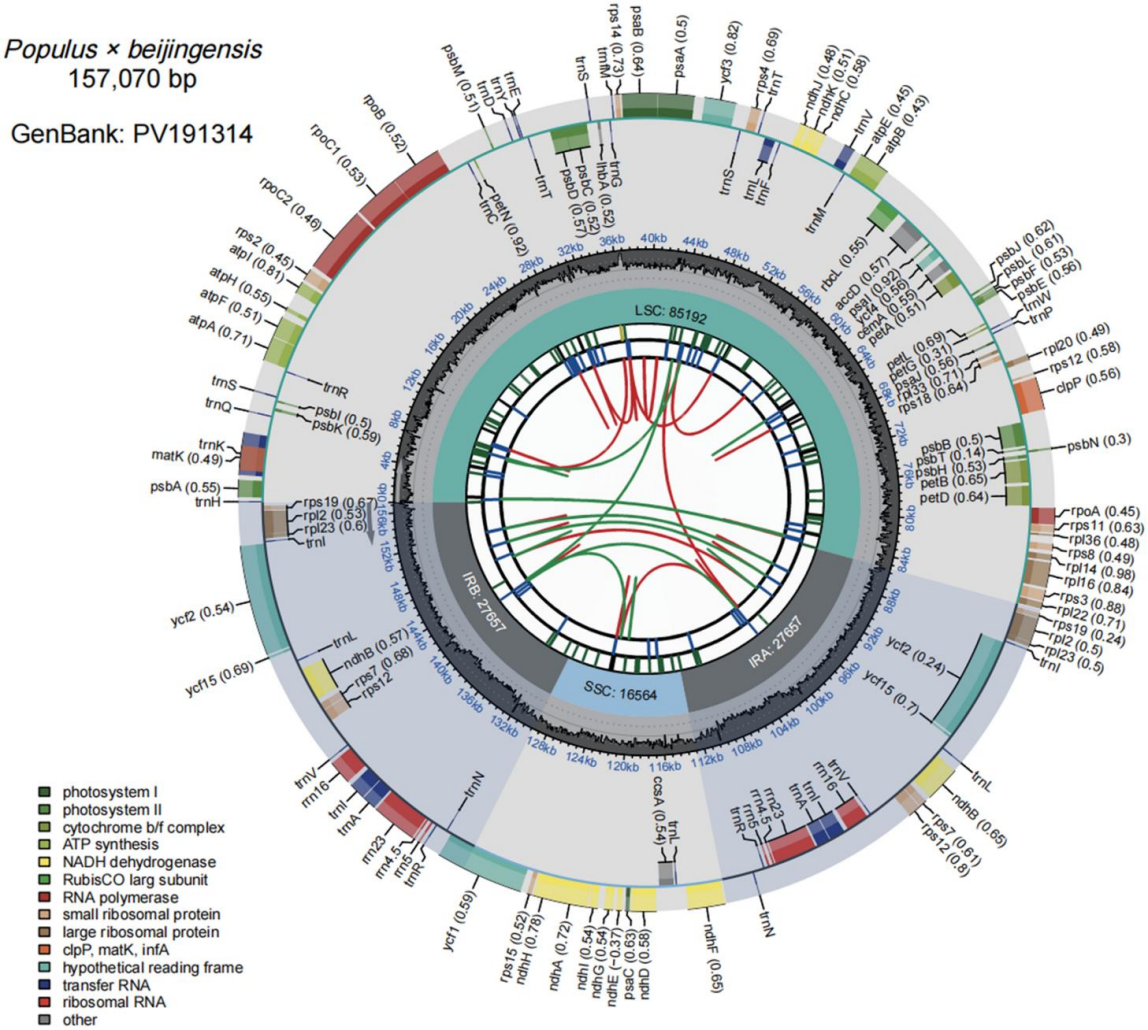
This schematic diagram generated using CPGView shows the overall characteristics of the complete chloroplast genome of *populus* × *beijingensis*. Different functional genes are identified by different colors, and their functions are displayed in the lower left corner. The innermost circle depicts dispersed repeats, including forward repeats (connected by red arcs) and palindrome repeats (connected by green arcs). The second circle explains the long tandem repeats represented by blue short bars. In the third circle, simple sequence repeats are represented by short colored bars, each bar indicating a different repeat unit size (RUS). The fourth circle depicts regions including small single copies (SSC), inverted repeat sequences (IRa and IRb), and large single copies (LSC). The fifth track plots the GC content of the entire plastid. In the sixth circle, the plastid genes are annotated, and the parentheses after each gene name indicate the optional codon usage bias. Genes are color-coded according to their functions, as shown in the legend. The genes in the inner and outer circles are transcribed clockwise and counterclockwise, respectively.

**Figure 3.**
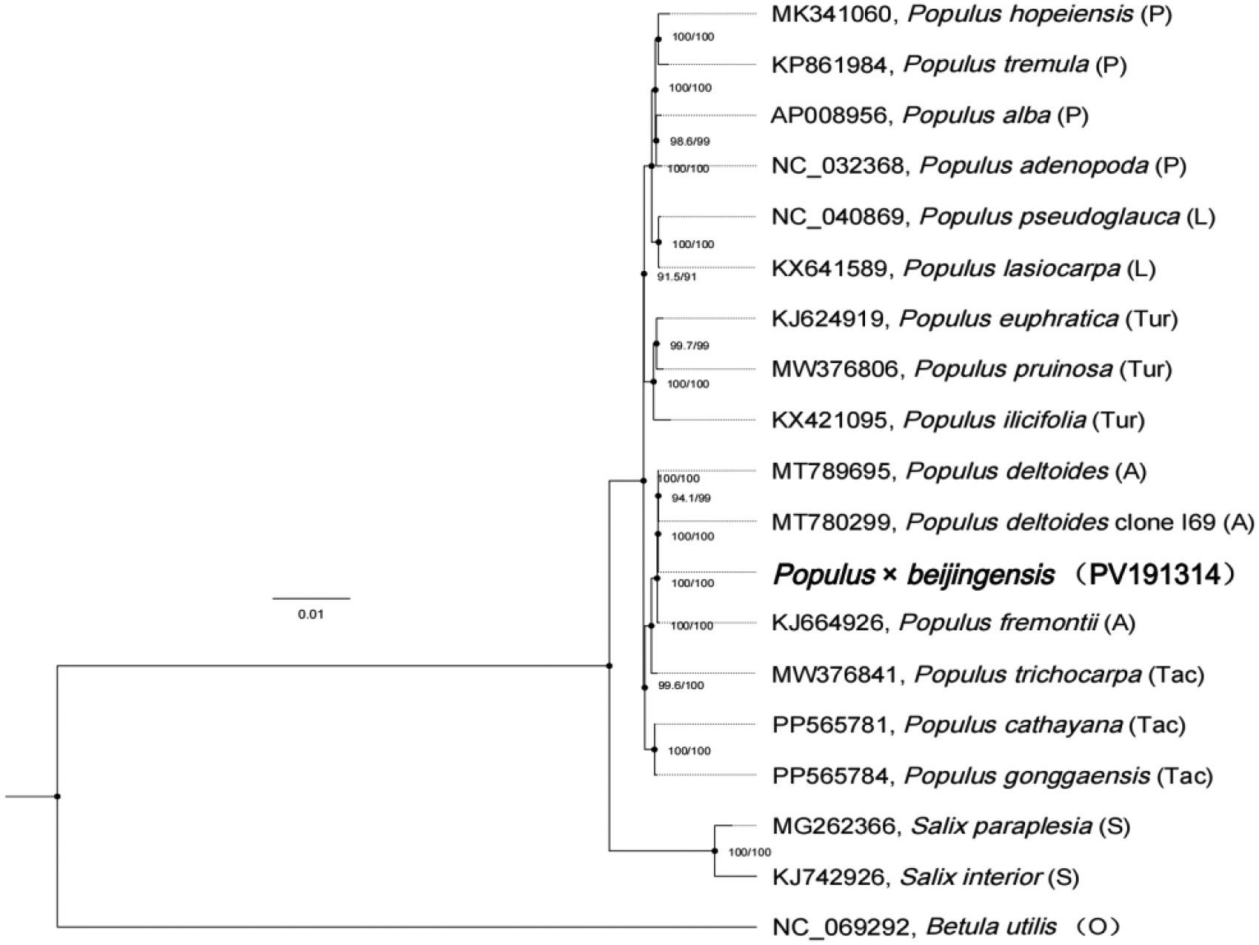
The maximum likelihood method was used to construct the phylogenetic tree of *populus × beijingensis* and other related species. Node numbers represent P, leuce; A, aigeiros; tac, tacamahaca; L, leucoides; tur, turanga; S, *salix*; O, outgroup. The preceding numbers appearing beside the branches represent the SH-like approximate likelihood ratio test, and the following numbers represent ultrafast bootstrap. The following sequences were used in this study: *Populus hopeiensis* MK341060 (Zong et al. 2019), *Populus tremula* KP861984 (Kersten et al. 2016), *Populus alba* AP008956 (Okumura et al. 2006), *Populus adenopoda* NC_032368, *Populus pseudoglauca* NC_040869 (Zong et al. 2019), *Populus lasiocarpa* KX641589, *Populus euphratica* KJ624919 (Zhang et al. 2016), *Populus pruinosa* MW376806 (Wang et al. 2022), *Populus ilicifolia* KX421095 (Chen et al. 2016), *Populus deltoides* MT789695 (Zhuang et al. 2020), *Populus deltoides* clone I69 MT780299 (Zhu et al. 2018), *Populus fremontii* KJ664926 (Huang et al. 2014), *Populus trichocarpa* MW376841 (Wang et al. 2022), *Populus cathayana* PP565781, *Populus gonggaensis* PP565784, *Salix paraplesia* MG262366, *Salix interior* KJ742926 (Huang et al. 2014), *Betula utilis* NC_069292.

The complete chloroplast genome of *P.×beijingensis* has been successfully assembled and annotated. The size of the chloroplast genome is 157,070 bp, which includes two inverted repeat regions (IRs), each spanning 27,657 bp. The large single-copy (LSC) region measures 85,192 bp, while the small single-copy (SSC) region is 16,564 bp. The total GC content of the *P.×beijingensis* chloroplast genome is 36.70%. This genome encodes a total of 128 genes, including 84 protein-coding genes, 36 transfer RNA (tRNA) genes, and 8 ribosomal RNA (rRNA) genes (Figure 2, Table S1). The sequencing depth values of *P.×beijingensis* have a maximum value of 5719×, a minimum value of 30×, and an average value of 3133.857× (Figure S1). The phylogenetic tree was constructed using 15 species of Populus, two species of Salix and *Betula utilis* as an outgroup (Table S2). The phylogenetic tree shows that *P.×beijingensis* belongs to the Aigeiros species within the *Populus,* and is highly similar to other tree species within the Aigeiros species, such as *Populus deltoides* clone I69, *Populus deltoides*, and *Populus fremontii*, forming a sister group (Figure 3).

By analyzing the chloroplast genomes of four species (*P.×beijingensis, Populus trichocarpa, Populus tomentosa, Populus alba* var*. Pyramidalis*) of poplar trees, it was found that there were certain differences in the genomic structure among these four species, for instance, the positions of genes such as *rpl22, trnN*, and *ndhF* were different (Figures S2 and S3). Ten genes involving cis-splicing (*atpF, rpoC1, ycf3, clpP, petB, petD, rpl16, rpl2, ndhB, ndhA*) and one gene involving trans-splicing (*rps12*) were identified (Figures S4 and S5).

## Discussion and conclusions

*Populus × beijingensis* is a new inter-specific hybrid variety developed by the Chinese Academy of Forestry through crossbreeding between the black poplar strain as the maternal parent and the green poplar strain as the paternal parent (Wang and Doung 1982). The genetic parent characteristics of *P.×beijingensis* are different from those of its parent poplar species. Compared to its parents, it has a wider geographical distribution and its adaptability is reflected in its extensive compatibility with soil, climate and water conditions, enabling it to grow and reproduce in various environmental conditions (Zhang et al. 2022; Luo et al. 2024).

*Populus × beijingensis* as a type of poplar tree produced through hybridization using black poplar as the parent, has enriched the variety of the Aigeiros (Li et al. 2004). Some studies based on the analysis of chloroplast fragment sequences have shown that the remaining tree species of the black poplar group form a monophyletic cluster with a 100% support rate. However, *P.×beijingensis*, due to its complete inheritance of the chloroplast genome from the mother plant, is separately clustered with the mother plant (Yuan et al. 2015).

This study is the first to obtain and analyze the complete sequence of the chloroplast genome of *P.×beijingensis*, and systematically evaluate its structural integrity and sequence fidelity. The highly variable regions such as trnR and ndhC are not only powerful molecular markers for the phylogeny of the *Populus*, but may also drive the environmental adaptation and hybridization infiltration of the *Populus* by regulating the photosynthetic stress network, tracing the maternal lineage background, and mediating nuclear-organellar co-evolution, providing theoretical basis and technical paths for the protection and precise breeding of *Populua* genetic resources.

## Supplementary Material

Supplementary files of Populus beijingensis clean copy.docx

## Data Availability

The genome sequence data that support the findings of this study are openly available in GenBank of NCBI at (https://www.ncbi.nlm.nih.gov/) under the accession no. PV191314. The accession no. of BioProject, Bio-Sample, and SRA are PRJNA1237998, SAMN47447914, and SRR32769322, respectively.
