# Supplementary material for: The complete chloroplast genome sequence of the populus cultivar “Populus × Beijingensis”: a type of unique Chinese populus
Source: Mitochondrial DNA B Resour. 2025 Sep 16;10(10):967–71. doi: 10.1080/23802359.2025.2559715 (PMC12444954; doi:10.1080/23802359.2025.2559715)
Supplement: Supplementary files of Populus beijingensis clean copy.docx [file TMDN_A_2559715_SM3424.docx]

**Table S1**. List of genes in chloroplast genome of *Populus* × *beijingensis*

| **Gene Functions** | **Group of genes** | **Name of genes** |
| --- | --- | --- |
| Photosynthesis | Subunits of ATP synthase  Subunits of NADH dehydrogenase  Subunits of cytochrome Subunits of photosystem  Subunits of photosystem II  Subunit of rubisco | *atpA*, *atpB*, *atpE*, *atpF**, *atpH*, *atpI*,  *ndhA**, *ndhB**(×2), *ndhC*, *ndhD*, *ndhE*, *ndhF*, *ndhG*, *ndhH*,*ndhI*, *ndhJ*, *ndhK*  *petA*, *petB* *, *petD**, *petG*, *petL*, *petN*  *psaA*, *psaB*, *psaC*,*psaJ*, *psaI*  *psbA*, *psbB*, *psbC*, *psbD*, *psbE*, *psbF*, *psbH*, *psbI*, *psbJ*, *psbK*, *psbL*, *psbM*, *psbN*, *psbT*  *rbcL* |
| Self-replication | Large subunit of ribosome  Small subunit of ribosome  DNA dependent RNA polymerase Ribosomal RNAs  Transfer RNAs | *rpl2**(×2), *rpl14*, *rpl16**, *rpl20*, *rpl22*, *rpl23*(×2), *rpl33*, *rpl36*  *rps2*, *rps3*, *rps4*, *rps7*(×2), *rps8*, *rps11*, *rps12***(×2) ,*rps14*, *rps15*, *rps18*, *rps19*(×2)  *rpoA*, *rpoB*, *rpoC1* *, *rpoC2*  *rrn4.5S*(×2), *rrn5S*(×2), *rrn16S*(×2), *rrn23S*(×2)  *trnA-UGC**(×2), *trnC-GCA*, *trnD-GUC*, *trnE-UUC*,  *trnF-GAA*, *trnG-UCC*, *trnH-GUG*, *trnI-CAU*(×2),  *trnI-GAU**(×2), *trnK-UUU**, *trnL-CAA*(×2), *trnL-UAA**, *trnL-UAG*, *trnM-CAU*, *trnN-GUU*(×2), *trnP-UGG*,  *trnQ-UUG*, *trnR-ACG*(×2), *trnR-UCU*, *trnS-GCU*, *trnS-GGA*, *trnS-UGA*, *trnT-GGU*, *trnT-UGU*,  *trnV-GAC*(×2), *trnV-UAC**, *trnW-CCA,* *trnY-GUA*, *trnfM-CAU* |
| Other genes | Maturase  Protease  Envelop membrane protein  Subunit of Acetyl-CoA-carboxylase c-type cytochrome synthesis gene  Translational initiation | *matK*  *clpP***  *cemA*  *accD*  *ccsA*  *infA* |
| Unknown function | Conserved open reading frames | *lhbA*, *ycf1*, *ycf2*(×2), *ycf3***, *ycf15*(×2) |

Note: *gene with a single intron; **gene with two introns; (×2) duplicated gene.

**Table S2**. Phylogenetic tree references

| **Species** | **GenBank** | **Reference** | **doi** |
| --- | --- | --- | --- |
| *Populus hopeiensis*  *Populus tremula*  *Populus alba*  *Populus pseudoglauca*  *Populus lasiocarpa Populus pruinosa*  *Populus adenopoda*  *Populus euphratica*  *Populus ilicifolia*  *Populus deltoides*  *Populus deltoides clone I69*  *Populus fremontii*  *Populus trichocarpa*  *Populus cathayana*  *Populus gonggaensis*  *Salix paraplesia*  *Salix interior*  *Betula utilis* | MK341060  KP861984  AP008956  NC_040869  KX641589  MW376806  NC_032368  KJ624919  KX421095  MT789695  MT780299  KJ664926  MW376841  PP565781  PP565784  MG262366  KJ742926  NC_069292 | Zong et al.  2019a  Kersten et al.  2016  Okumura et al.  2006  Zong et al.  2019b  Wang et al.  2022  Zhang et al.  2016  Chen et al.  2016  Zhuang et al.  2020 Zhu et al. 2018  Huang et al.  2014 Wang et al.2022  Huang et al.  2014 | 10.1371/journal.pone.0218455  10.1371/journal.pone.0147209 10.1007/s11248-006-9009-3  10.3389/fpls.2019.00005 Unpublished  10.3389/fpls.2022.813177 Unpublished  10.3109/19401736.2014.913159  10.1007/s12686-016-0566-3  10.1080/23802359.2020.1833773 10.1186/s12864-018-4813-8  10.1111/nph.12956  10.3389/fpls.2022.813177 Unpublished  Unpublished  Unpublished  10.1111/nph.12956  Unpublished |

References

Chen Z, Wang W, Yang W, Ma T.2016. Characterization of the complete chloroplast genome of *Populus ilicifolia*.

*Conservation Genetics Resources*. 8(4):1-3. doi:10.1007/s12686-016-0566-3.

Huang DI, Hefer CA, Kolosova N, Douglas CJ, Cronk QCB. 2014. Whole plastome sequencing reveals deep plastid divergence and cytonuclear discordance between closely related balsam poplars, *Populus*

*balsamifera* and *P.trichocarpa* (*Salicaceae*). *New Phytologist*. 204(3):693-703. doi:10.1111/nph.12956.

Kersten B, Faivre Rampant P, Mader M, Le Paslier MC, Bounon R, Berard A, Vettori C, Schroeder H, Leplé JC,

Fladung M. 2016. Genome Sequences of *Populus tremula* Chloroplast and Mitochondrion: Implications for

Holistic Poplar Breeding. *Public Library of Science ONE*. 11(1):e0147209. doi:10.1371/journal.pone.

Okumura S, Sawada M, Park YW, Hayashi T, Shimamura M, Takase H, Tomizawa K. 2006. Transformation of poplar

(*Populus alba*) plastids and expression of foreign proteins in tree chloroplasts. *Transgenic Res*. 15(5):637-46.

doi:10.1007/s11248-006-9009-3.

Wang Y, Huang J, Li E, Xu S, Zhan Z, Zhang X, Yang Z, Guo F, Liu K, Liu D, Shen X, Shang C, Zhang Z. 2022.

Phylogenomics and biogeography of *Populus* based on comprehensive sampling reveal deep-level relationships and multiple intercontinental dispersals. *Frontiers In Plant Science*. 13:813177. doi:10.3389/fpls.2022.813177.

Zhang QJ, Gao LZ. 2016. The complete chloroplast genome sequence of desert poplar (*Populus euphratica*).

*Mitochondrial DNA A DNA Mapp Seq Anal*. 27(1):721-3. doi:10.3109/19401736.2014.913159.

Zhuang W, Shu X, Zhang M, Wang T, Zhang F, Wang N, Wang Z. 2020. Characterization of the complete chloroplast genome of *Populus deltoides* Zhonglin 2025. *Mitochondrial DNA B Resour*. 5(3):3723-3724.

doi:10.1080/23802359.2020.1833773.

Zong D, Gan P, Zhou A, Li J, Xie Z, Duan A, He C. 2019a. Comparative analysis of the complete chloroplast genomes of seven *Populus* species: Insights into alternative female parents of *Populus tomentosa*. *Public Library of*

*Science ONE.* 14(6):e0218455. doi:10.1371/journal.pone.0218455.

Zong D, Gan P, Zhou A, Zhang Y, Zou X, Duan A, Song Y, He C. 2019b. Plastome sequences help to resolve deep-level

relationships of *Populus* in the family salicaceae. *Frontiers In Plant Science*. 10:5. doi:10.3389/fpls.2019.00005.

Zhu S, Xu M, Wang H, Pan H, Wang G, Huang M. 2018. Study of spontaneous mutations in the transmission of poplar

chloroplast genomes from mother to offspring. *BMC Genomics*. 19(1):411. doi: 10.1186/s12864-018-4813-8


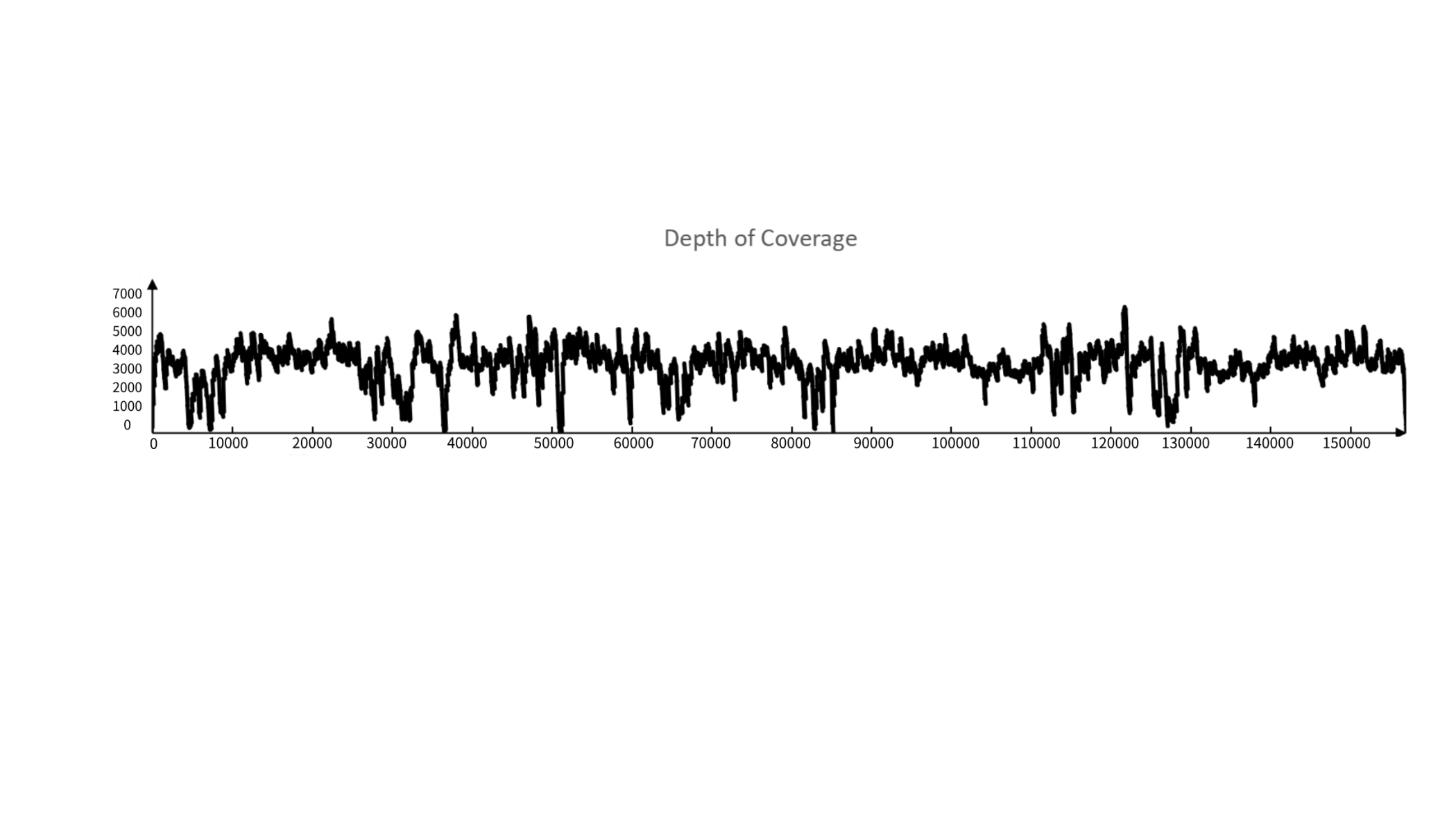


**Figure S1.** The coverage depth of the chloroplast genome of *Populus×beijingensis.* The sequencing depth values of *P.×beijingensis* have a maximum value of 5719×, a minimum value of 30×, and an average value of 3133.857×.


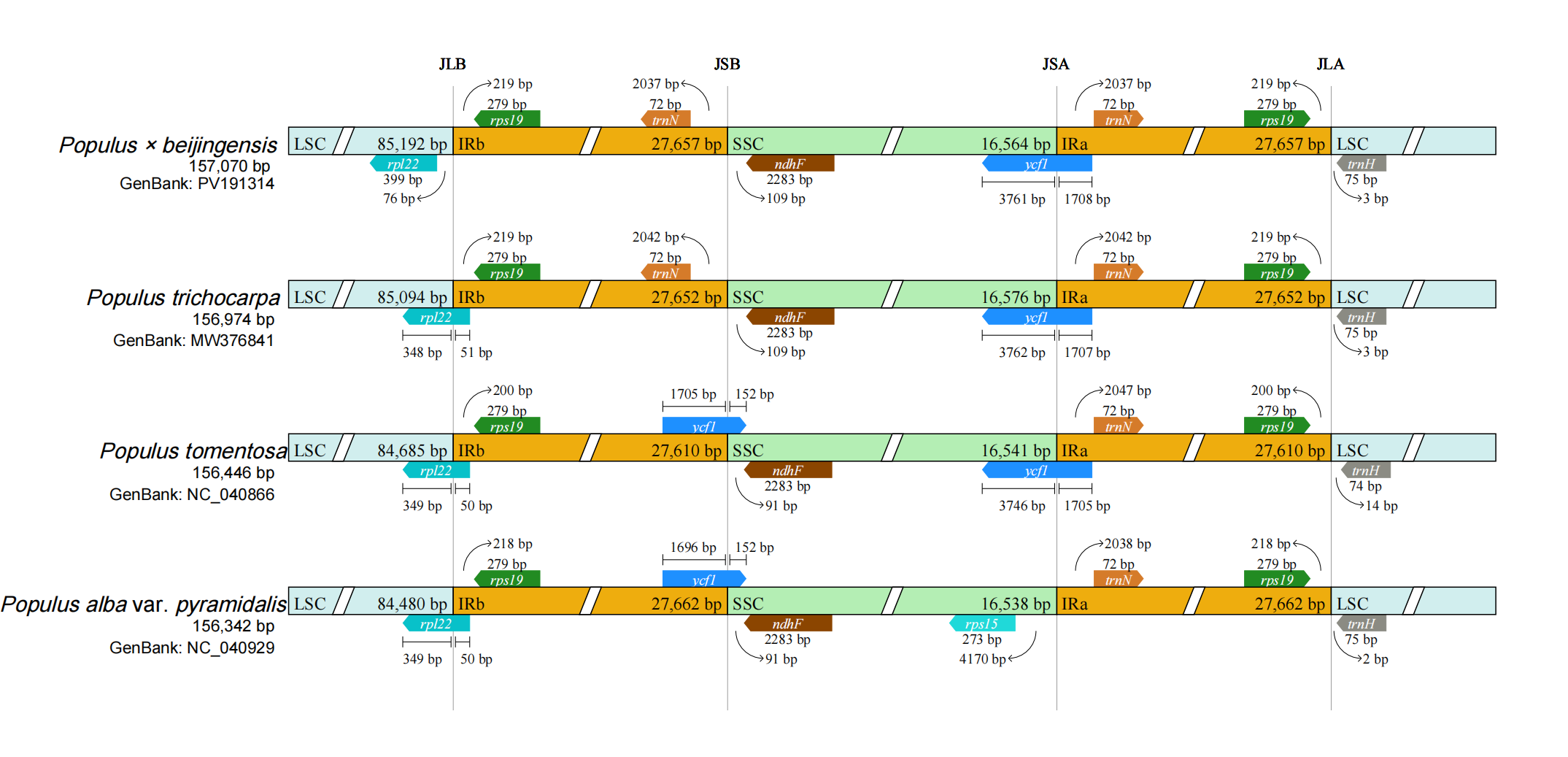


**Figure S2.** The physical map of the chloroplast genome of *Populus×beijingensis*. It was constructed by comparing *Populus×beijingensis* with *Populus trichocarpa, Populus tomentosa*, and *Populus alba* var*. Pyramidalis*. The locations of multiple genes (such as *rpl22, trnN*, and *ndhF*) vary among the four species of the *Populus*.


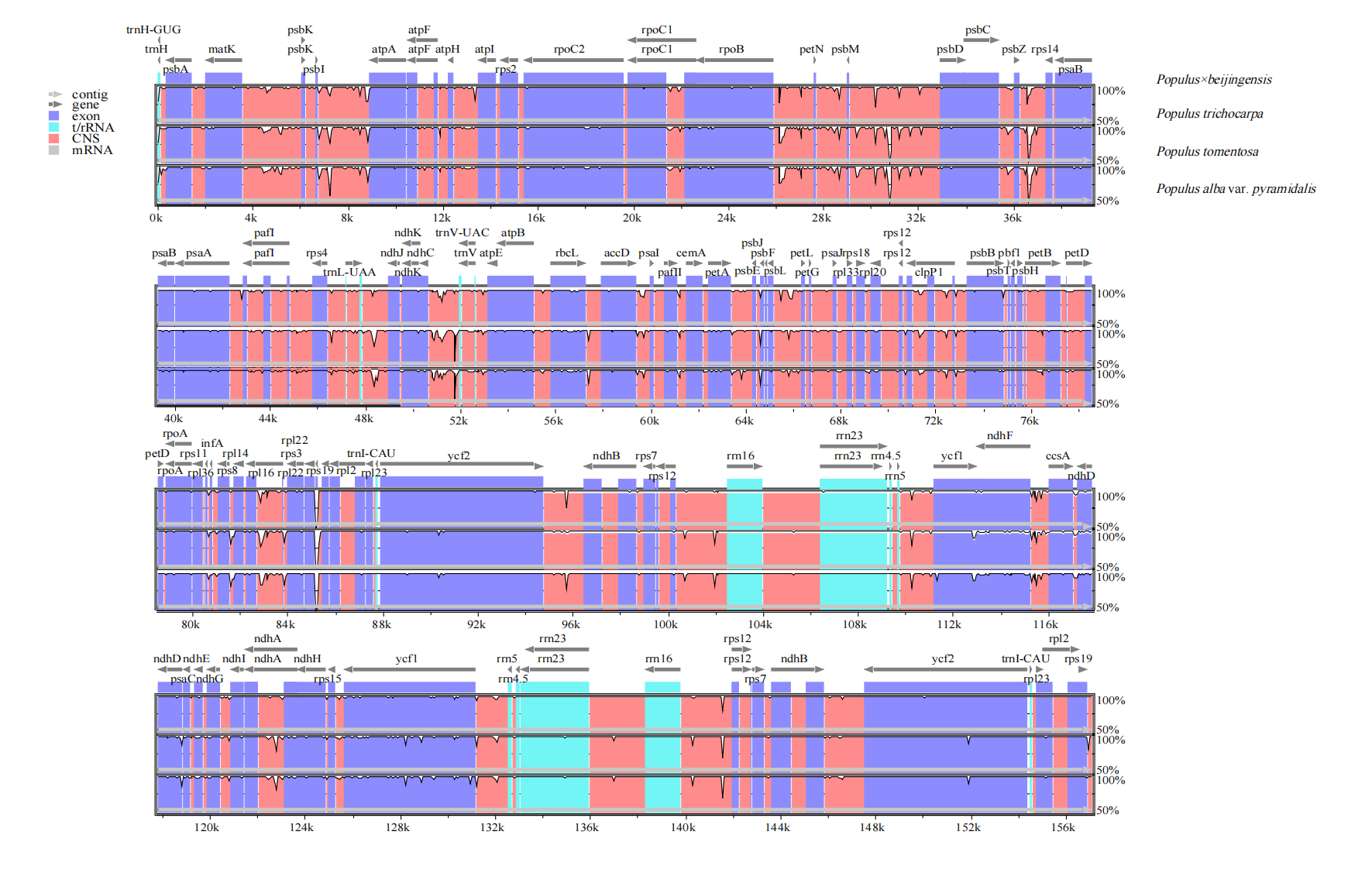


**Figure S3.** mVISTA analysis of *Populus×beijingensis*, *Populus trichocarpa* (GenBank: MW376841),

*Populus tomentosa* (GenBank: NC_040866), and *Populus alba* var. *pyramidalis* (GenBank: NC_

040929).


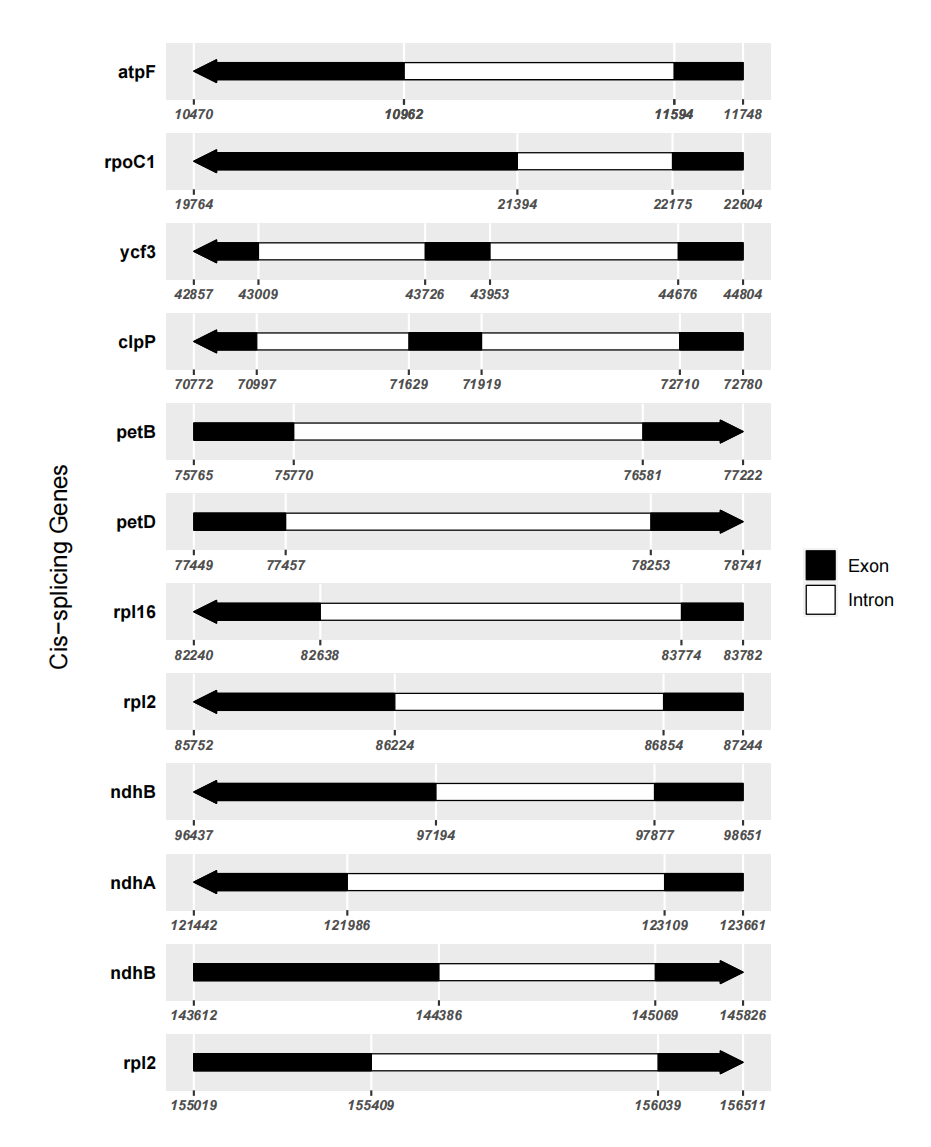


**Figure S4.** Schematic map of the cis-splicing genes in the chloroplast genome of *Populus×beijingensis*. Exons and introns are shown in black and white, respectively. The arrow indicates the sense direction of the gene.


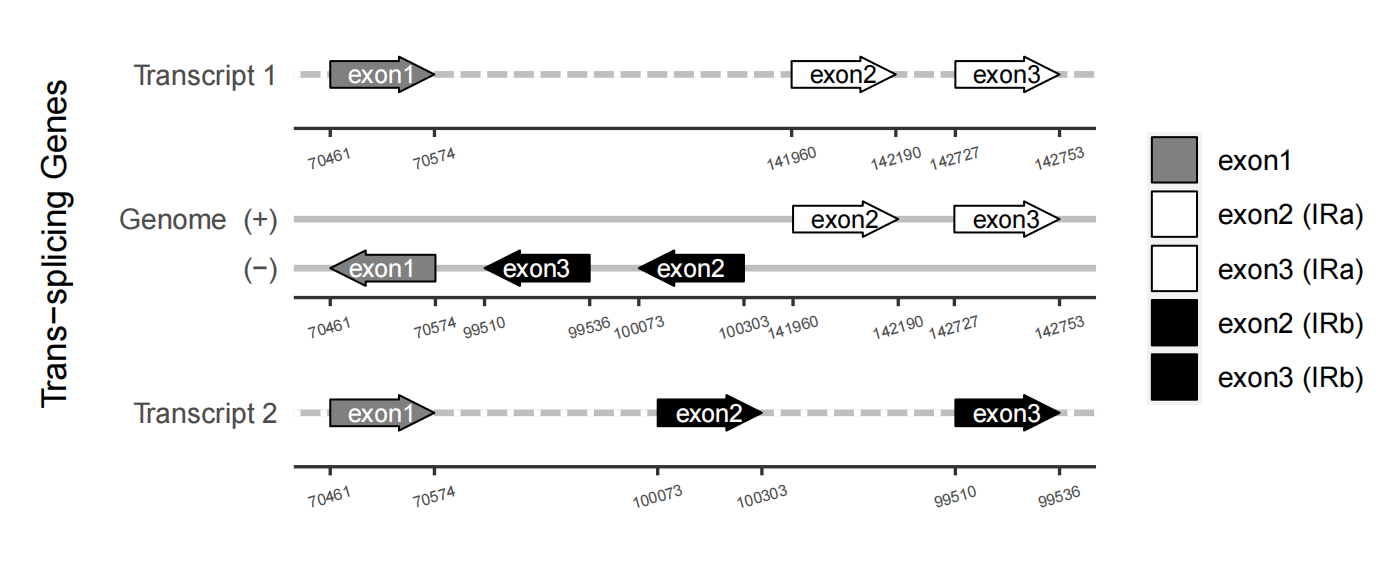


**Figure S5.** Schematic map of the trans-splicing gene *rps12* in the chloroplast genome of *Populus×beijingensis.*
